# Dental Treatment Needs and Cost Burden Among Older Adults: A K-Means Cluster Analysis to Inform Oral Health Policies

**DOI:** 10.3390/ijerph23060797

**Published:** 2026-06-14

**Authors:** Burcu Aksoy, Şükrü Can Akmansoy, Yasemin Özkan, Gonca Mumcu

**Affiliations:** 1 Medical Promotion and Marketing Program, Department of Medical Services and Techniques, Vocational School, Istanbul Atlas University, Istanbul 34408, Türkiye; 2 Department of Prosthodontics, Faculty of Dentistry, Marmara University, Istanbul 34854, Türkiye; can.akmansoy@marmara.edu.tr (Ş.C.A.); ykozkan@marmara.edu.tr (Y.Ö.); 3 Department of Oral and Maxillofacial Radiology, Faculty of Dentistry, Istanbul Okan University, Istanbul 34959, Türkiye; gonca.mumcu@okan.edu.tr

**Keywords:** aged, oral health, health services needs, health care costs, cluster analysis

## Abstract

**Highlights:**

**Public health relevance—How does this work relate to a public health issue?**

- Oral health problems in older adults represent a growing public health concern due to ageing populations and increasing treatment needs.
- This study examines treatment patterns and cost burden in older adults, highlighting inequalities in access to and utilization of dental care.

**Public health significance—Why is this work of significance to public health?**

- The study identifies that relatively younger older adults experience higher treatment complexity and cost burden, indicating unmet preventive care needs.
- It provides evidence linking oral health conditions with economic burden, emphasizing the importance of early prevention strategies.

**Public health implications—What are the key implications for practitioners, policymakers and/or researchers?**

- Results support the implementation of preventive and age-specific oral health programmes to reduce long-term treatment costs.
- Results also offer evidence for policymakers to improve resource allocation and develop sustainable oral health policies for ageing populations.

**Abstract:**

Oral health problems among older adults represent a growing public health concern due to increasing life expectancy and treatment needs. This study aimed to assess dental treatment needs and cost burden within the context of oral health policies. This retrospective study included anonymized data from 250 patients aged ≥65 years (F/M: 121/129; 65–89 years). Sociodemographic characteristics, treatment needs, and costs were obtained from the Hospital Information Management System (HIMS). Costs were adjusted to 2025 Turkish lira values using the Consumer Price Index and converted to international dollars using purchasing power parity (PPP). Patients were classified by total treatment costs using K-means cluster analysis. Periodontal (61.2%), restorative (36.0%), and endodontic (41.2%) treatment needs, which are largely preventable through oral hygiene practices, were more frequent among patients with a lower mean age, whereas tooth loss and prosthodontic treatment needs (89.6%) increased with mean age. Cluster analysis identified two groups: a low-cost group (67.6%) and a high-cost group (32.4%). The high-cost group had a lower mean age (68.84 ± 4.27 years) compared to the low-cost group (70.73 ± 5.18 years), indicating that relatively younger patients needed more complex and costly treatments. Out-of-pocket payments were notable for prosthodontic and surgical treatments, although Social Security Institution (SSI) payments constituted most of the costs. Preventive and early dental care strategies are essential to reduce treatment complexity and cost burden among older adults within the framework of oral health policy.

## 1. Introduction

Ageing is a multidimensional process characterized by progressive physiological changes, declining functional capacity, and the coexistence of chronic diseases [1]. Owing to the global increase in life expectancy, the proportion of older adults in the population is rising rapidly worldwide, making healthy ageing a major public health priority [2,3]. Oral health is inevitably affected by the ageing process and represents an essential component of overall health and quality of life. Oral health problems and tooth loss may impair chewing, eating, speaking, and social interaction. They also negatively affect both the physical and psychosocial well-being and quality of life of older adults [4,5]. Poor oral health is associated with malnutrition, frailty and reduced quality of life among ageing populations [6,7].

Older adults are particularly vulnerable to oral health problems because of systemic diseases, polypharmacy, reduced manual dexterity, cognitive decline, and barriers to accessing dental services [7,8]. Furthermore, periodontal disease, dental caries and tooth loss—all of which increase treatment complexity and cost—are highly prevalent among older adults [4,5,9,10,11]. In addition, delayed treatment-seeking behaviours contribute to unmet oral health needs in older adults [8]. Similar results have been reported in Türkiye. National and regional studies have indicated that older adults in Türkiye have high Decayed, Missing, Filled Teeth (DMFT) scores, substantial tooth loss, and limited utilization of preventive applications [12]. Many older adults visit dentists only when symptoms or pain occur, while routine dental check-ups remain insufficient in Türkiye [13,14]. Furthermore, periodontal diseases, tooth loss, and systemic diseases among older adults in Türkiye have been associated with poorer oral health-related quality of life, as well as reduced physical activity and nutritional status [15]. In addition, prosthodontic treatment needs are highly prevalent among geriatric patients because of extensive tooth loss and inadequate oral hygiene practices [16]. Socioeconomic inequalities, limited oral health literacy, and financial barriers may further worsen oral health outcomes and restrict access to dental care among older adults in Türkiye [17]. Therefore, protecting oral health is a critical priority for both older adults and oral health policies targeting ageing populations [9,11,12,18]. Poor oral health contributes to increased treatment costs within the scope of oral health policies [19].

The increasing oral healthcare needs of ageing populations also create a significant economic burden for both individuals and healthcare systems [11,19]. Older adults may experience difficulties in accessing dental care because of high treatment costs, transportation barriers, and functional limitations [8,20]. Although public healthcare systems provide care for older adult patients [21], out-of-pocket payments often represent a significant portion of dental care expenditures [11,19]. This financial burden poses a significant barrier to access for older adults with limited resources [10]. Consequently, evidence-based oral health policies and preventive strategies are essential to reduce treatment needs, improve accessibility, and support healthy ageing [12,22].

In this context, the use of K-means cluster analysis has increasingly expanded in healthcare studies because it is an unsupervised machine learning approach for identifying hidden patterns within heterogeneous patient populations. By grouping individuals according to shared clinical characteristics and healthcare utilization profiles, clustering methods may support population segmentation and facilitate the evaluation of treatment complexity, disease burden, and healthcare utilization patterns [23,24]. K-means-based analyses are particularly useful for patients with chronic diseases and multimorbidity for identifying subgroups with different healthcare needs, resource utilization patterns and treatment cost profiles [25,26]. In addition, clustering approaches may contribute to more efficient healthcare planning by identifying clinically meaningful patient profiles within large and heterogeneous healthcare datasets [23]. Finally, clustering approaches may provide valuable insights into oral health policy by identifying older adult patients with different treatment complexities and treatment-related cost burden profiles.

Despite the growing importance of geriatric oral healthcare, to our knowledge, dental treatment needs with cost burden among older adults have not been previously evaluated in studies conducted in Türkiye. In addition, although clustering methods are increasingly used in healthcare research, studies using K-means cluster analysis to identify high-cost patient profiles and evaluate treatment-related costs among older adults remain limited. From clinical and public health perspectives, understanding treatment complexity and cost burden in ageing populations is important for preventive care planning, efficient resource allocation, and sustainable oral health policy development. Therefore, this retrospective study aimed to assess dental treatment needs and treatment-related cost burden among older adults and to identify cost-based patient profiles using K-means cluster analysis within the context of oral health policy.

## 2. Materials and Methods

This retrospective study was performed by using anonymous data saved on HIMS in a public dental school, Marmara University Istanbul, Türkiye. The study included data from 250 older adults aged ≥65 years (F/M:121/129; mean age: 70.12 ± 4.98 years; 65–89 years) whose treatments were completed between September 2018 and March 2020. This period was selected to ensure access to comprehensive and up-to-date data following the update of the HIMS database, while avoiding potential disruptions in healthcare service delivery associated with the onset of the COVID-19 pandemic.

All patients aged ≥65 years were included in the study using the census approach. Patients whose dental treatments were fully completed and whose payment records in HIMS were complete within the predefined study period were included in the study. Therefore, no formal sample size calculation or a priori power analysis was performed.

Data on the patient’s sociodemographic profile (age, gender, marital status), treatment needs, and their treatment costs according to SSI payments and out-of-pocket payments were used in the study.

In the analysis, dental treatment needs were grouped into main categories based on the departments in which they were provided. Radiological examinations included diagnostic imaging procedures such as panoramic radiographs, periapical radiographs, and computed tomography. Periodontal treatments included procedures such as scaling, which involves the removal of dental plaque and calculus from tooth surfaces, and curettage, which refers to the removal of inflamed soft tissue within periodontal pockets. Restorative treatments included procedures aimed at restoring the structure and function of teeth affected by caries or other defects, primarily through tooth fillings. Endodontic treatments involved the management of pulpal and periapical diseases through root canal treatment. Prosthodontic treatments covered removable and fixed dentures. Surgical treatments included tooth extraction and other oral surgical procedures [5,27]. This classification was used to ensure consistency in the analysis and to allow comparison of treatment needs across patients.

Age was classified according to the World Health Organization [28], into three groups: 65–74 years, 75–84 years, and ≥85 years. Because the ≥85 years age group (*n* = 6, 2.4%), included a very small number of participants, patients aged ≥75 years were combined into a single category for statistical analyses to avoid unstable subgroup comparisons. Accordingly, for the analysis of treatment costs, age was categorized into two groups (65–74 years and ≥75 years) to facilitate interpretation and comparison of cost differences between age groups. In this study, “younger patients” refers to individuals aged 65–74 years, while “older patients” refers to those aged ≥75 years.

Data on treatment cost for each dental procedure were obtained from the HIMS records at the procedure level. Procedure-level costs recorded in Turkish lira were grouped according to the related dental treatment categories, including radiological examinations, periodontal, restorative, endodontic, prosthodontic, and surgical treatments. For each patient, procedure-level costs were aggregated within each treatment category, and SSI payments and out-of-pocket payments were calculated separately for dental treatment category. All relevant SSI and out-of-pocket cost items in HIMS were incorporated into the cost analysis for each treatment category.

To ensure comparability over time and reflect current price levels, for each treatment category, SSI payments and out-of-pocket payments recorded in Turkish lira were separately adjusted to 2025 price levels using the Consumer Price Index published by the Turkish Statistical Institute [29]. Following inflation adjustment, SSI and out-of-pocket cost components were separately converted into purchasing power parity international dollars (PPP US$) using the most recent available World Bank PPP conversion factor at the time of analysis, corresponding to the year 2024. Accordingly, the conversion was performed using a rate of 1 international dollar = 11.55 Turkish liras. After PPP conversion, SSI and out-of-pocket costs were summed to obtain the total treatment cost for each treatment category. Finally, the PPP-adjusted total costs of all treatment categories were combined to calculate the overall total treatment cost for each patient. This overall patient-level total treatment cost was used as the cost variable in the K-means cluster analysis. The PPP US$, as defined by the World Bank, reflects the amount of local currency required to purchase the same quantity of goods and services as one US dollar would in the United States. Therefore, the use of PPP conversion enabled the standardization and international comparability of treatment costs across different treatment categories [30,31].

The study was approved by the Ethics Committees of the Institute of Health Science of Marmara University (approval number: 115; date of approval: 18 October 2021).

### 2.1. Statistical Evaluation

Data were analyzed by using SPSS 29.0 statistic software (IBM Corp., Armonk, NY, USA). Mann–Whitney U test was applied owing to the non-normal distribution of data. Categorical data were presented as “*n*” and “%” in tables. Chi-square test was used in their analysis. This analysis revealed significant patterns among treatments across different dental departments, indicating that patients frequently received multiple types of treatments simultaneously. Thereafter, K-means cluster analysis was conducted to classify total treatment costs into homogeneous groups and to determine the relationships among variables by identifying patterns of cost distribution across related treatment combinations. Since the total cost data showed a non-parametric distribution with numerous outliers, the total costs were standardized using Z-scores prior to clustering. This transformation minimized the influence of extreme values and ensured the reliability of the K-means algorithm, which is sensitive to mean-based calculations [32,33]. In the study, *p* < 0.05 was accepted as statistically significant.

### 2.2. K-Means Cluster Analysis

Cluster analysis aims to reveal similarities among observations according to their specific characteristics and to classify them into homogeneous groups based on these similarities. K-means cluster analysis was used as a common unsupervised machine learning approach to evaluate complex data structures and identify patterns among variables. K-means clustering is a partitioning algorithm that classifies observations into clusters by minimizing within-cluster variance and maximizing between-cluster separation. The algorithm iteratively assigns observations to the nearest cluster centroid and updates cluster centres until cluster similarity is optimized. K-means clustering groups data points into a predetermined number of clusters based on similarity characteristics. This method is particularly useful for identifying hidden patterns and grouping structures within numerical healthcare data. In addition, clustering approaches may support patient stratification, healthcare planning, and the identification of subgroups with varying treatment complexities and healthcare burden profiles [23,24,25,26,32,33]. Therefore, K-means clustering was considered appropriate for identifying relatively homogeneous patient groups according to treatment-related cost characteristics. In the present study, K-means cluster analysis was performed to identify patient groups with similar characteristics according to total treatment costs. The optimal number of clusters was initially explored using the elbow method (R version 4.5.2; R Foundation for Statistical Computing, Vienna, Austria). The two-cluster solution was considered the most appropriate for identifying clinically meaningful treatment-cost patterns among older adults and was retained for the final analysis.

## 3. Results

The sociodemographic profile of the study group and distribution of patient assessment and treatment needs are presented in Table 1. In the study group (*n* = 250), the mean age was 70.12 ± 4.98 years, 51.6% of patients were male, and 75.2% were married. Most patients were aged 65–74 years (*n* = 211, 84.4%), while 15.6% were aged ≥75 years (*n* = 39, 15.6%).

In addition to clinical examinations, patients were also examined in the Department of Oral and Maxillofacial Radiology by using different imaging techniques (98.4%). The highest treatment needs were observed in departments regarding Prosthodontics (89.6%) and Periodontology (61.2%). Oral and Maxillofacial Surgery (55.2%) and Endodontics (41.2%) were also commonly seen in older adult patients. Additionally, more than one-third of patients needed Restorative Dentistry treatment (36.0%). No statistically significant associations were found between gender or marital status and treatment needs (*p* > 0.05).

When the relationship between age and treatment needs was examined, patients requiring treatment from the departments of Periodontology (69.36 ± 4.75 years), Restorative Dentistry (69.16 ± 4.70 years), and Endodontics (68.36 ± 3.45 years) had lower mean ages than other patients who did not need these treatments (71.31 ± 5.12 years; 70.66 ± 5.06 years; 71.35 ± 5.50 years, respectively) (*p* < 0.001; *p* = 0.005; *p* < 0.001). In contrast, similar associations were not observed for Oral and Maxillofacial Radiology examination and treatments needs in Prosthodontics, and Oral and Maxillofacial Surgery (*p* > 0.05) (Table 2).

Furthermore, patients who needed multidisciplinary care across all dental departments had a significantly lower mean age (68.38 ± 4.13 years) compared to those who needed treatment from four or fewer departments (70.45 ± 5.06 years; *p* = 0.003) (Table 2).

Statistically significant differences were observed in mean age based on specific dental treatment needs. Patients who needed scaling (59.6%, 69.42 ± 4.76 years), curettage (57.2%, 69.45 ± 4.76 years), tooth filling (36.4%, 69.00 ± 4.41 years), partial denture (42.4%, 69.42 ± 4.87 years), and crown (51.2%, 68.86 ± 4.27 years) treatments had a lower mean age than those who did not need these respective treatments (*p* < 0.05 for all comparisons). In addition, patients who received complete denture treatment (50.8%, 71.05 ± 5.55 years) due to complete tooth loss had a higher mean age than patients who did not receive complete denture (49.2%, 69.15 ± 4.10 years) (*p* = 0.005). In contrast, no significant differences in age were observed for implant-supported partial denture (2.6%, 72.67 ± 8.77 years vs. 97.6%, 70.05 ± 4.86 years) and implant-supported crown treatments (2.0%, 67.80 ± 3.03 vs. 98%, 70.16 ± 5.00) (*p* > 0.05), because they were performed in only a limited number of patients.

Significant associations were found among treatments received in different departments. Patients who needed periodontal treatment were significantly more likely to also need restorative (54.2%), endodontic (62.7%), and surgical treatments (67.3%) (*p* < 0.001). Similarly, endodontic treatment was commonly associated with prosthodontic (95.1%) and surgical treatments (67.0%) (*p* < 0.05), indicating that these interventions are often performed together. In addition, surgical treatments were significantly associated with all other departments (*p* < 0.05), suggesting their important role in comprehensive dental care. Based on the significant co-occurrence of treatments in the chi-square analysis, patients were subsequently stratified by age for further cost analyses.

The total treatment cost across all departments was higher among younger patients (aged 65–74 years) (1463.42 ± 1173.38 PPP US$) compared to older patients (aged ≥75 years) (1129.34 ± 795.94 PPP US$). Moreover, the highest treatment cost in both age groups was prosthodontic treatment. This was followed by endodontic and periodontal treatments, respectively (Table 3).

Patients were classified into two clusters representing low-cost and high-cost groups based on total treatment costs using the K-means clustering method for subsequent analyses. Cluster analysis was used to identify distinct cost burden profiles among older adults rather than to predict outcomes, providing a practical framework for resource planning in older adults’ dental care. Cluster-1 (*n* = 169, 67.6%), with a mean of 822.17 ± 478.49 PPP US$ in total treatment costs, was defined as the “Patient Group with Low Total Treatment Cost”, while Cluster-2 (n = 81, 32.4%), with a mean of 2640.48 ± 1103.27 PPP US$ in total treatment costs, was defined as the “Patient Group with High Total Treatment Cost”. The patients in Cluster-2 had a lower mean age (68.84 ± 4.27 years) than those of Cluster-1 (Table 4). Moreover, gender and marital status were not significantly associated with cost-based patient clusters identified by K-means cluster analysis (*p* > 0.05).

When SSI payments in clusters were evaluated according to departments, these payments were higher for Oral and Maxillofacial Radiology, Endodontics, and Prosthodontics departments in Cluster-2 than those of Cluster-1 (*p* < 0.001; *p* < 0.001; *p* < 0.001). Additionally, when out-of-pocket payments were also evaluated, patients in Cluster-2 had higher out-of-pocket payments for Oral and Maxillofacial Radiology, Prosthodontics, and Oral and Maxillofacial Surgery compared to those in Cluster-1 (*p* = 0.007; *p* < 0.001; *p* = 0.012) (Table 4).

Radiological examinations regarding panoramic film (96.4%) in Cluster-1 and (96.3%) in Cluster-2 were shown to be an essential method in clinical practice. The distribution of treatments performed in the departments is presented for both clusters in Table 5. The most common treatments were complete denture (63.3%), scaling (44.4%), and curettage (42.0%) in Cluster-1. On the other hand, Cluster-2 exhibited different trends; the most common treatments were crown (92.6%), scaling (91.4%), curettage (82.9%), and root canal treatment (79.0%) (Table 5).

## 4. Discussion

Demographic data on oral health problems are essential for planning effective and cost-efficient healthcare services, and access to evidence-based data supports the development of effective health policies [4]. Identifying treatment needs and associated costs among older adults is particularly important for understanding service demand and guiding oral health policy development, especially in the context of unmet needs arising from high treatment costs [22].

In the present study, the ratio of Oral and Maxillofacial Radiological examination was found to be high because diagnostic examinations using different imaging techniques were essential for treatment planning in patients. In contrast, the lowest treatment need was observed in Restorative Dentistry. More than half of the patients in the study group needed periodontal treatment, while a significant proportion received endodontic treatment. Similar results have been reported in previous studies, where periodontal disease and dental caries were associated with higher unmet dental needs in older adults [6,27]. A study in Australia also indicated that older adults have broad treatment needs encompassing restorative, preventive, prosthodontic, endodontic, surgical, and periodontal treatments, suggesting that preventable oral health problems persist in this population [11].

These results suggest that oral health problems are commonly seen among older adults, consistent with previous epidemiological studies reporting high prevalence of periodontal disease, dental caries, and tooth loss in ageing populations [6,11,34]. Given the effects of ageing on oral health [9], these results may indicate inadequate oral hygiene practices and limited access to dental care during ageing. From a gerodontology perspective, routine screening and preventive interventions should be integrated into primary healthcare services for older adults because this approach may help reduce complex treatment needs. Furthermore, low oral health literacy may contribute to insufficient utilization of dental care services among older adults. Therefore, strengthening educational programmes may help reduce future treatment needs.

In the study group, the majority of older adults needed prosthodontic (89.6%) treatment, and more than half needed surgical treatments (55.2%). These results were consistent with previous studies demonstrating that prosthodontic treatment constitutes the primary dental treatment need among older adult populations due to extensive tooth loss [7,21,35,36,37]. Surgical interventions are often required prior to prosthodontic treatment, increasing treatment complexity and cost [27,34]. Prosthodontic treatment needs were clinically relevant among older adults, as tooth loss is associated with impaired mastication, nutritional deficiencies, and poor quality of life owing to functional limitation and esthetic problems [9,27,34,35,38].

Age-related differences were observed in treatment needs. Periodontal, restorative, and endodontic treatment needs, as well as crown restorations and partial dentures were common among patients with a lower mean age. In contrast, the need of complete dentures was prevalent due to tooth loss among patient with a higher mean age. Consistent with the literature, older adults were more likely to be completely edentulous [8]. This pattern aligns with the classification of older adult populations into “young-old” (65–74 years), “middle-old” (75–84 years), and “oldest-old” (≥85 years), and reflects the progressive nature of oral health deterioration with age [37].

Similar results have been reported in a population-based study conducted in Iran, where oral health status was evaluated across different older age groups, and both DMFT scores and prevalence of severe tooth loss increased significantly with advancing age [5]. Prevalence of periodontal disease could increase with age [34] and unmet treatment needs are also higher among individuals aged 65–69 years. Treatment needs in Periodontal, Restorative and Endodontic departments arise from poor oral hygiene [5,27,39]. In the study group, 16.0% of patients needed complex treatments across all departments, and had a lower mean age than others. This supports the relationship between oral health problems and age as previously shown [27,40]. Furthermore, the reported decline in dental treatments during the COVID-19 pandemic period supports the exclusion of pandemic-affected treatment records in the present study to minimize potential disruptions in healthcare utilization patterns [16].

These results may indicate that relatively younger older adults tend to retain more natural teeth requiring restorative and periodontal care, whereas advancing age is associated with increased tooth loss and greater need for prosthodontic treatments. Overall, these results reflected the progressive deterioration of oral health with age. Therefore, age-specific differences should be considered in planning of dental services for older adults.

Treatment cost analysis performed using PPP revealed that total treatment costs were higher among younger patients (aged 65–74 years) compared with older patients (aged ≥75 years), with a significant difference observed only in restorative treatment costs. Elevated treatment costs among relatively younger older adults may reflect the retention of natural teeth. The need for complex and high-cost treatments among younger older adults may also reflect delayed utilization of dental services during early adulthood.

In addition, differences in healthcare utilization patterns, treatment-seeking behaviour, and access to dental services may also have contributed to the observed cost differences between age groups [19,41]. These are particularly important within the framework of healthy ageing policies, as preserving natural dentition and oral function during the early stages of ageing may reduce nutritional problems, and healthcare expenditures.

In the present study, K-means cluster analysis enabled the identification of distinct low-cost and high-cost patient profiles among older adults. Consistent with previous cluster studies, the identified clusters demonstrated substantial heterogeneity in resource utilization, supporting the usefulness of clustering approaches for identifying clinically meaningful patient subgroups [25,32]. A notable result was that the high-cost group was predominantly composed of relatively younger older adults. This trend may suggest that preventive oral healthcare interventions are not being sufficiently utilized before advanced treatment needs emerge, potentially leading to increased multidisciplinary treatment requirements during the early stages of ageing. Furthermore, SSI payments exceeded out-of-pocket payments, particularly prosthodontic and endodontic treatments. These results have important implications for oral health policy.

Therefore, expanding preventive oral health programmes may provide long-term economic benefits not only for individuals but also for public health financing systems. This issue is likely to become increasingly important in Türkiye due to population ageing. On the other hand, out-of-pocket payments were higher for radiological examinations, prosthodontic, and surgical treatments, potentially increasing the financial burden on older adults. Overall, these results suggest that age-related differences in treatment complexity and cost patterns should be considered in oral healthcare planning. Policy strategies such as community-based oral screening programmes and mobile dental services for older adults with limited mobility, and integration of oral health assessments into routine geriatric care may help to reduce long-term treatment burden.

From a health management perspective, cluster-based stratification supports patient prioritization, risk-based service planning, and the development of targeted recall systems [23,24]. Since cluster-based stratification may facilitate the prioritization of patients who are more likely to develop complex multidisciplinary treatment needs, this approach could potentially lead to a more efficient allocation of resources and the integration of oral health assessments into routine geriatric care.

These results also showed the importance of preventive applications in reducing both treatment needs and associated costs. Previous studies have shown that early interventions are less costly than complex restorative and prosthodontic treatments [42,43,44,45,46]. Global cost analyses using PPP further highlight the significant economic burden of oral diseases [47]. Finally, limited access to oral healthcare services may further exacerbate these burdens.

Older adults represent a vulnerable population due to socioeconomic disadvantages, chronic conditions, and mobility limitations that restrict access to oral healthcare [8,20,48]. These results may support the integration of gerodontology-focused approaches into oral healthcare planning [9,38,49,50]. From this perspective, routine oral examinations, dental and periodontal monitoring, prosthodontic maintenance, and individualized preventive counselling should be incorporated into regular care for older adults. In addition, regular oral health screening and treatment of oral health problems at an early stage may help to reduce treatment complexity and tooth loss during ageing. Preventive care-oriented strategies may help decrease treatment burden among older adults. Public oral healthcare systems may also benefit from risk-based patient stratification approaches targeting relatively younger older adults with complex multidisciplinary treatment needs. Expanding community-based preventive oral health programmes and strengthening gerodontology-focused training for dental professionals may further contribute to reducing long-term treatment burden and healthcare expenditures associated with ageing populations.

This study provides valuable insights into treatment needs and cost burden among older adult patients attending a public dental school in Istanbul. However, several limitations should be considered when interpreting the results. First, the study was conducted retrospectively using HIMS data obtained from a single institutional setting during a specific time period and using a census-based, non-probabilistic approach. Therefore, the results primarily reflect the characteristics of patients attending this public dental hospital and may not be fully generalizable to the broader older adult population. In addition, treatment-related costs were calculated in patients whose treatments were completed during the predefined study period and payment records were available in HIMS according to the census approach. These may be thought of as selection bias by potentially excluding individuals with interrupted treatment processes, more severe health conditions, limited financial access to care, or restricted healthcare utilization. Therefore, the external validity and generalizability of the results are limited and context specific.

Another limitation of this study was that the data were collected between September 2018 and March 2020. This period was selected to ensure consistency, and completeness of the HIMS records as well as to avoid disruptions associated with the onset of the COVID-19 pandemic. Changes in oral healthcare utilization, healthcare access and treatment patterns after the pandemic may limit the current relevance and generalizability of the results. Nevertheless, the study provided valuable evidence regarding treatment complexity, cost structure and public financing burden in a tertiary public dental care setting. Institution-based data nevertheless provided valuable information for dentalcare planning and resource allocation.

A further limitation concerned the age classification strategy used in the analysis. Combining participants aged ≥75 years into a single category may reduce comparability with studies that use the full World Health Organization age classification (65–74, 75–84, and ≥85 years). However, this approach was necessary due to the very small number of participants in the ≥85 years group, which would otherwise result in unstable estimates and reduced statistical reliability. In particular, the very limited number of participants aged ≥85 years reduced the representativeness of the “oldest-old” population and limited the generalizability of results for this age group, which is known to experience substantial oral health problems, tooth loss, and complex treatment needs.

The other limitation was that socioeconomic profile and income-related information were not in HIMS in the study. Treatment preferences may be influenced by financial affordability, treatment duration and the number of required clinical visits. Therefore, the potential influence of socioeconomic factors on treatment choices could not be evaluated in the present study. This limitation should be considered while interpreting the results.

Finally, treatment costs were derived from a dental institution of a public university operating within the SSI reimbursement system. Therefore, the reported costs reflect public-sector treatment pricing and reimbursement policies. They may differ substantially from costs in private dental clinics, where minimum price tariff systems and out-of-pocket payment structures vary. In addition, some dental treatment options may not be fully covered or routinely provided within public institutions.

## 5. Conclusions

Dental treatment needs and treatment-related cost burden were found to be different according to age-related oral health profiles among older adults in this study. Treatment needs from Periodontal, Restorative, and Endodontic departments were more common among relatively younger adults, whereas complete tooth loss and prosthodontic treatment needs were observed in older age groups.

K-means cluster analysis identified two distinct patient profiles based on total treatment costs. The high-cost group had a lower mean age compared with the low-cost group. These results suggested age-related differences in treatment patterns and treatment-related cost burden among older adults.

From a health management perspective, the results highlighted the importance of strengthening preventive and early intervention strategies for older adults. Improving oral health literacy and increasing regular dental visits may help to reduce treatment complexity, tooth loss and long-term economic burden. In addition, the substantial contribution of SSI payments to treatment costs indicated the need for sustainable resource allocation and evidence-based planning within public oral healthcare services.

Overall, this study provided evidence that preventive, age-sensitive, and patient-centred oral health approaches are essential to support healthy ageing. Multicenter and longitudinal studies including socioeconomic variables are recommended to better evaluate oral healthcare access, treatment preferences, and long-term cost burden among older adults in future.

## Figures and Tables

**Table 1:** Sociodemographic profile of the study group and distribution of patient assessment and treatment needs.

	Mean	SD
**Age (years)**	70.12	4.98
	***n***	**%**
**Gender**		
Female	121	48.4
Male	129	51.6
Total	250	100
**Marital Status**		
Married	188	75.2
Single	59	23.6
Unknown	3	1.2
Total	250	100
**Patient Assessment and Treatment Needs**		
Oral and Maxillofacial Radiology	246	98.4
Periodontology	153	61.2
Restorative Dentistry	90	36.0
Endodontics	103	41.2
Prosthodontics	224	89.6
Oral and Maxillofacial Surgery	138	55.2

SD: Standard deviation.

**Table 2:** Relationship between age and treatment needs.

		Age (Years)		
	*n* (%)	Mean	SD	*p*
**Oral and Maxillofacial Radiology**				
Yes No	246 (98.4) 4 (1.6)	70.15 68.00	5.00 2.58	0.464
**Periodontology**				
Yes No	153 (61.2) 97 (38.8)	69.36 71.31	4.75 5.12	**<0.001**
**Restorative Dentistry**				
Yes No	90 (36.0) 160 (64.0)	69.16 70.66	4.70 5.06	**0.005**
**Endodontics**				
Yes No	103 (41.2) 147 (58.8)	68.36 71.35	3.45 5.50	**<0.001**
**Prosthodontics**				
Yes No	224 (89.6) 26 (10.4)	70.07 70.54	5.04 4.45	0.382
**Oral and Maxillofacial Surgery**				
Yes No	138 (55.2) 112 (44.8)	70.04 70.21	5.27 4.60	0.395
**Patients Receiving Treatment from All Departments (Periodontology, Restorative Dentistry, Endodontics, Prosthodontics, Oral and Maxillofacial Surgery)** **Patients Receiving Treatment from a Maximum of Four Departments**	40 (16.0) 210 (84.0)	68.38 70.45	4.13 5.06	**0.003**

SD: Standard deviation. The Mann–Whitney U test was used for comparisons. Bold values indicate statistically significant differences (*p* < 0.05).

**Table 3:** Distribution of total treatment costs by age groups.

	Costs (PPP US$)			
	65–74 Years		≥75 Years	
	Mean	SD	Mean	SD
**Total Treatment Costs**	1463.42	1173.38	1129.34	795.94
**Departments**				
Oral and Maxillofacial Radiology	38.42	28.35	31.34	25.41
Periodontology	169.04	95.26	138.22	49.77
Restorative Dentistry *	123.76	111.25	54.16	25.68
Endodontics	203.73	150.75	146.77	73.26
Prosthodontics	1179.12	800.81	1044.94	602.85
Oral and Maxillofacial Surgery	157.18	414.73	123.63	171.08

PPP US$: Purchasing power parity international dollars. SD: Standard deviation. The Mann–Whitney U test was used for comparisons. * *p* = 0.008.

**Table 4:** Comparison of age and total dental treatment costs between patient clusters identified by K-means cluster analysis.

	Cluster-1 Patient Group with Low Total Treatment Cost (PPP US$) (*n* = 169, 67.6%)	Cluster-2 Patient Group with High Total Treatment Cost (PPP US$) (*n* = 81, 32.4%)	*p*
	Mean ± SD	Mean ± SD	
**Age (years)**	70.73 ± 5.18	68.84 ± 4.27	**0.002**
**Total Treatment Costs**	822.17 ± 478.49	2640.48 ± 1103.27	**<0.001**
**SSI Payments**			
Oral and Maxillofacial Radiology	20.63 ± 10.97	28.20 ± 15.03	**<0.001**
Periodontology	146.89 ± 74.44	179.09 ± 100.94	0.057
Restorative Dentistry	106.50 ± 100.61	114.64 ± 83.55	0.289
Endodontics	14.63 ± 12.09	28.80 ± 14.37	**<0.001**
Prosthodontics	414.22 ± 291.25	1222.54 ± 470.86	**<0.001**
Oral and Maxillofacial Surgery	62.55 ± 59.41	81.76 ± 96.35	0.205
**Out-of-Pocket Payments**			
Oral and Maxillofacial Radiology	29.85 ± 25.66	39.00 ± 23.81	**0.007**
Periodontology	132.46	71.89 ± 52.08	0.800
Restorative Dentistry	49.71 ± 92.27	33.01 ± 31.31	0.530
Endodontics	63.63 ± 52.32	88.73 ± 70.57	0.317
Prosthodontics	336.65 ± 196.68	713.39 ± 670.56	**<0.001**
Oral and Maxillofacial Surgery	129.68 ± 174.07	828.82 ± 888.23	**0.012**

PPP US$: Purchasing power parity international dollars. SSI: Social Security Institution. SD: Standard deviation. The Mann–Whitney U test was used for comparisons. Bold values indicate statistically significant differences (*p* < 0.05).

**Table 5:** Distribution of treatment procedures applied in departments.

	Cluster-1 Patient Group with Low Total Treatment Cost (PPP US$) (*n* = 169)		Cluster-2 Patient Group with High Total Treatment Cost (PPP US$) (*n* = 81)	
Treatment Procedures	n	%	n	%
Panoramic Film	163	96.4	78	96.3
Vitality Control	31	18.3	54	66.7
Periapical Film	35	20.7	42	51.9
Dental Tomography	13	7.6	11	13.5
Scaling	75	44.4	74	91.4
Curettage	71	42.0	72	82.9
Filling	42	24.9	49	60.5
Root Canal Treatment	38	22.5	64	79.0
Crown	53	31.4	75	92.6
Temporomandibular Joint Examination	68	40.2	58	71.6
Complete Denture	108	63.3	19	22.2
Partial Denture	46	27.2	60	74.1
Tooth Extraction	57	33.1	51	63.0

PPP US$: Purchasing power parity international dollars.

## Data Availability

The data that support the findings of this study are available from Marmara University Dental School. Restrictions apply to the availability of these data, which were used under licence for this study. Data are available from the author(s) with the permission of Marmara University Dental School.

## Abbreviations

The following abbreviations are used in this manuscript:

DMFT	Decayed, Missing, Filled Teeth
HIMS	Hospital Information Management System
SSI	Social Security Institution
PPP	Purchasing Power Parity
